# Supplementary material for: Association between air quality index and effects on emergency department visits for acute respiratory and cardiovascular diseases
Source: PLoS One. 2023 Nov 16;18(11):e0294107. doi: 10.1371/journal.pone.0294107 (PMC10653395; doi:10.1371/journal.pone.0294107)
Supplement: S1 File — (DOCX) [file pone.0294107.s001.docx]

**Supplementary Material**

**Daily air quality index calculation of each type of air pollution substance**

Calculated from the concentration of air pollution from the air quality measurement results with the level of air pollution concentration that is equivalent to the air quality index at various levels as follows (Table 1). Calculation of air quality index within the level is a straight line equation as follows.

I_p_ = [(I_HI_ - I_LO_)/(BP_HI_ - BP_LO_) * (C_p_ - BP_LO_)] + I_LO_

Where,

I_p_ = Index for pollutant p.

I_HI_ = AQI value corresponding to BP_HI._

I_LO_ =AQL value corresponding to BP_LO._

BP_HI_ = Breakpoint that is greater than or equal to C_p_.

BP_LO_ =Breakpoint that is less than or equal to C_p_.

C_p_ = Rounded concentration of pollutant p.

The highest index (highest value of I_p_) represents the AQI of the location.

**Table Air pollution concentration equivalent to air quality index**

| **AQI Cluster** | **PM_2.5_ (µg./m^3^)** | **PM_10_ (µg./m^3^)** | **O_3_ (ppb)** | **CO (ppm)** | **NO_2_ (ppb)** | **SO_2_ (ppb)** |
| --- | --- | --- | --- | --- | --- | --- |
|  | **Average 24 hours continuously.** | | **Average 8 hours continuously.** | | **Average 1 hour** | |
| 0 - 25 | 0 - 25 | 0 - 50 | 0 - 35 | 0 - 4.4 | 0 - 60 | 0 - 100 |
| 26 - 50 | 26 - 37 | 51 - 80 | 36 - 50 | 4.5 - 6.4 | 61 - 106 | 101 - 200 |
| 51 - 100 | 38 - 50 | 81 - 120 | 51 - 70 | 6.5 - 9.0 | 107 - 170 | 201 - 300 |
| 101 - 200 | 51 - 90 | 121 - 180 | 71 - 120 | 9.1 - 30.0 | 171 - 340 | 301 - 400 |
| More than 200 | 91 and up | 181 and up | 121 and up | 30.1 and up | 341 and up | 401 and up |

µg./m3; Micrograms per cubic meter, ppb; parts per billion, ppm; parts per million

**Reference**

1. Air4Thai [Internet]. [cited 2023 Jul 22]. Available from: http://air4thai.pcd.go.th/webV2/aqi_info.php

2. Raj H, Vijaykumar S. A Study on Air Quality Index. ijcse [Internet]. 2019 [cited 2023 Jul 22];7:961–6. Available from: <http://www.ijcseonline.org/full_paper_view.php?paper_id=3947>

**Supplementary Table 1. Daily Air Ambient Data for the Study Period (April 2018 to March 2019)**

| **Air Pollutions** | **Mean (SD)** |  | **Percentiles** |  |  |
| --- | --- | --- | --- | --- | --- |
|  |  | **Median** | **Min** | **Max** | **IQR** |
| PM _2.5_ (µg/m^3^) | 89.0(40.2) | 75 | 39 | 282 | 63-102 |
| PM _10_ (µg/m^3^) | 45.2(21.2) | 39 | 9 | 176 | 32-53 |
| O_3_ (µg/m^3^) | 19.9(7.3) | 19 | 2 | 53 | 15-24 |
| NO_2_ (µg/m^3^) | 10.1(6.3) | 8 | 3 | 45 | 6-12 |
| SO_2_ (µg/m^3^) | 0.4(1.6) | 0 | 0 | 27 | 0-0 |

**
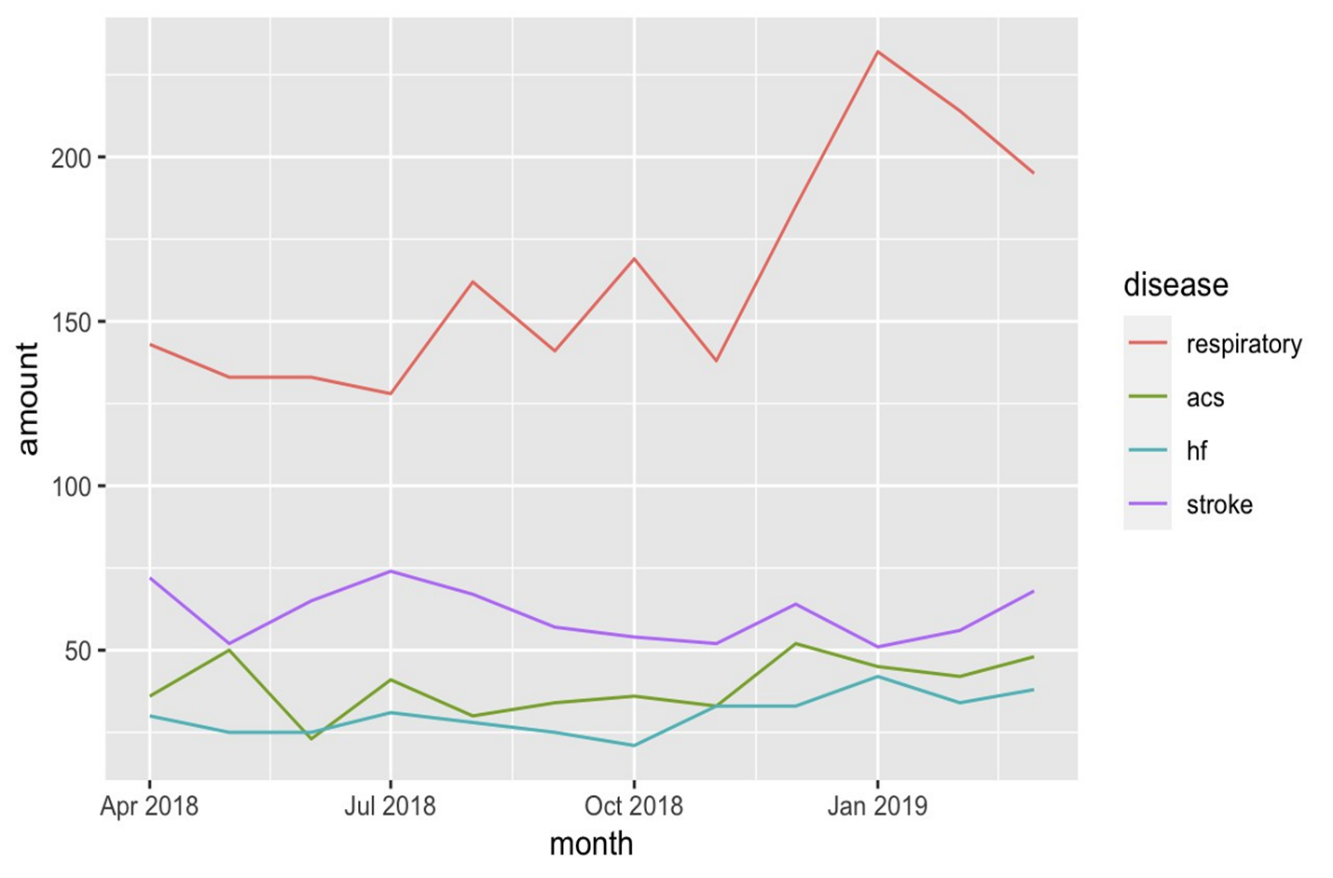
**

**Supplementary Figure 1. Seasonal Effect of PM_2.5_ Concentration with Specific Disease Variation**

**During the Study Period (April 2018 to March 2019)**

**Supplementary Figure 2. Variation of Each Air Pollutant Throughout the Study Period**

**(April 2018 to March 2019)**

**Supplementary Figure 3. Relative Risk of the Adjusted Lag-Effect between PM_10_ and ED Visits of Acute Respiratory Disease, ACS, AHF, Stroke, Pneumonia and COPD and Asthma**

**(Reference PM_10_ = 120 µg/m^3^)**

**Supplementary Figure 4. Excess Risk (95% Confident Intervals) of Association between Air Pollutants (PM_2.5,_ PM_10_, O_3_ and NO_2_) with ICU Admission**

**for Serious Specific Disease at any Lag Day**

**Supplementary Figure 5. Excess Risk (95% Confident Intervals) of Association between Air Pollutants (PM_2.5,_ PM_10_, O_3_ and NO_2_) with In-Hospital Death**

**for Serious Specific Disease at any Lag Day**
