# Supplementary material for: Association between air quality index and effects on emergency department visits for acute respiratory and cardiovascular diseases
Source: PLoS One. 2023 Nov 16;18(11):e0294107. doi: 10.1371/journal.pone.0294107 (PMC10653395; doi:10.1371/journal.pone.0294107)

PLOS ONE

Association Between PM 2.5 and Effect of Emergency Department Visits for Acute Respiratory Disease, Acute Coronary Syndrome, Acute Heart Failure, and Stroke

--Manuscript Draft--

**Manuscript Number:**	PONE-D-22-17550
**Article Type:**	Research Article
**Full Title:**	Association Between PM 2.5 and Effect of Emergency Department Visits for Acute Respiratory Disease, Acute Coronary Syndrome, Acute Heart Failure, and Stroke
**Short Title:**	PM 2.5 and Effect of Emergency Department Visits
**Corresponding Author:**	Borwon Wittayachmnankul<br>Chiang Mai University<br>Muang Chiang Mai, Chiang Mai THAILAND
**Keywords:**	Air pollutants; Environmental health; Emergency Department; Public Health; Pneumonia
**Abstract:**	Background and objective<br><br>To determine the association of PM2.5 and other pollutants effects on Emergency Department (ED) visits, hospitalization, and mortality with acute respiratory disease, acute coronary syndrome (ACS), acute heart failure (AHF), and stroke.<br><br>Methods<br><br>This study was a retrospective study with daily data collected on ED visits between 2018 and 2019 at Maharaj Nakorn Chiang Mai Hospital. A distributed lag non-linear model and quasi-Poisson model were used to explore the relationship between air quality parameters and ED visits in each disease.<br><br>Results<br><br>A total of 3,540 ED visits were recorded during study period. The mean daily PM2.5 concentration was 89.0±40.2 µg/m3. We observed associations between PM2.5 concentrations and the ED visits due to ACS on the following day (RR = 1.023, 95% confidence interval [CI]: 1.002-1.044) and two days after exposure (RR = 1.026, 95% CI: 1.005-1.047). Also, subgroup analysis revealed the association between PM2.5 concentrations and the ED visits due to pneumonia on the current day (RR =1.071, 95% CI: 1.025-1.118) and on the following day after exposure (RR =1.024, 95% CI: 1.003-1.046). PM2.5 associated with increased mortality resulted from ACS on lag day 3 (OR = 1.36, 95% CI: 1.08-1.73). For other air pollutions, PM10 is associated with increased ED visits due to COPD/asthma and increased hospitalization in AHF. Also, O3 and NO2 are associated with increased ICU admissions and mortality in AHF.<br><br>Conclusion<br><br>Short-term exposure to PM2.5 pollution can increase the risk of ED visits in ACS and acute respiratory disease particularly for pneumonia.
**Order of Authors:**	Panumas Surit, MD
	Wachira Wongtanasarasin, MD
	Chiraphat Boonnag, MD
	Borwon Wittayachmnankul
**Additional Information:**	
**Question**	**Response**
**Financial Disclosure**	Faculty of Medicine, Chiang Mai University funded this research (Fund No. 089-2563).

Powered by Editorial Manager® and ProduXion Manager® from Aries Systems Corporation

Enter a financial disclosure statement that describes the sources of funding for the work included in this submission. Review the submission guidelines for detailed requirements. View published research articles from *PLOS ONE* for specific examples.<br><br>This statement is required for submission and **will appear in the published article** if the submission is accepted. Please make sure it is accurate.<br><br>**Unfunded studies**<br>Enter: *The author(s) received no specific funding for this work.*<br><br>**Funded studies**<br>Enter a statement with the following details:<br>• Initials of the authors who received each award<br>• Grant numbers awarded to each author<br>• The full name of each funder<br>• URL of each funder website<br>• Did the sponsors or funders play any role in the study design, data collection and analysis, decision to publish, or preparation of the manuscript?<br>• **NO** - Include this sentence at the end of your statement: *The funders had no role in study design, data collection and analysis, decision to publish, or preparation of the manuscript.*<br>• **YES** - Specify the role(s) played.<br><br>* typeset	
**Competing Interests**<br><br>Use the instructions below to enter a competing interest statement for this submission. On behalf of all authors, disclose any competing interests that could be perceived to bias this work—acknowledging all financial support and any other relevant financial or non-financial competing interests.<br><br>This statement is required for submission and **will appear in the published article** if the submission is accepted. Please make	The authors have declared that no competing interests exist.

sure it is accurate and that any funding sources listed in your Funding Information later in the submission form are also declared in your Financial Disclosure statement.<br><br>View published research articles from *PLOS ONE* for specific examples.<br><br>**NO authors have competing interests**<br><br>Enter: *The authors have declared that no competing interests exist.*<br><br>**Authors with competing interests**<br><br>Enter competing interest details beginning with this statement:<br><br>*I have read the journal's policy and the authors of this manuscript have the following competing interests: [insert competing interests here]*<br><br>* typeset	
**Ethics Statement**<br><br>Enter an ethics statement for this submission. This statement is required if the study involved:<br><br>• Human participants<br>• Human specimens or tissue<br>• Vertebrate animals or cephalopods<br>• Vertebrate embryos or tissues<br>• Field research<br><br>Write "N/A" if the submission does not require an ethics statement.<br><br>General guidance is provided below. Consult the submission guidelines for detailed instructions. **Make sure that all information entered here is included in the Methods section of the manuscript.**	N/A

**Format for specific study types**<br><br>**Human Subject Research (involving human participants and/or tissue)**<br>• Give the name of the institutional review board or ethics committee that approved the study<br>• Include the approval number and/or a statement indicating approval of this research<br>• Indicate the form of consent obtained (written/oral) or the reason that consent was not obtained (e.g. the data were analyzed anonymously)<br><br>**Animal Research (involving vertebrate animals, embryos or tissues)**<br>• Provide the name of the Institutional Animal Care and Use Committee (IACUC) or other relevant ethics board that reviewed the study protocol, and indicate whether they approved this research or granted a formal waiver of ethical approval<br>• Include an approval number if one was obtained<br>• If the study involved *non-human primates*, add *additional details* about animal welfare and steps taken to ameliorate suffering<br>• If anesthesia, euthanasia, or any kind of animal sacrifice is part of the study, include briefly which substances and/or methods were applied<br><br>**Field Research**<br><br>Include the following details if this study involves the collection of plant, animal, or other materials from a natural setting:<br>• Field permit number<br>• Name of the institution or relevant body that granted permission	
**Data Availability**<br><br>Authors are required to make all data underlying the findings described fully available, without restriction, and from the time of publication. PLOS allows rare exceptions to address legal and ethical concerns. See the PLOS Data Policy and FAQ for detailed information.	Yes - all data are fully available without restriction

A Data Availability Statement describing where the data can be found is required at submission. Your answers to this question constitute the Data Availability Statement and **will be published in the article**, if accepted.<br><br>**Important:** Stating ‘data available on request from the author’ is not sufficient. If your data are only available upon request, select ‘No’ for the first question and explain your exceptional situation in the text box.<br><br>Do the authors confirm that all data underlying the findings described in their manuscript are fully available without restriction?	
**Describe where the data may be found in full sentences. If you are copying our sample text, replace any instances of XXX with the appropriate details.**<br><br>• If the data are **held or will be held in a public repository**, include URLs, accession numbers or DOIs. If this information will only be available after acceptance, indicate this by ticking the box below. For example: *All XXX files are available from the XXX database (accession number(s) XXX, XXX.).*<br>• If the data are all contained **within the manuscript and/or Supporting Information files**, enter the following: *All relevant data are within the manuscript and its Supporting Information files.*<br>• If neither of these applies but you are able to provide **details of access elsewhere**, with or without limitations, please do so. For example:<br><br>*Data cannot be shared publicly because of [XXX]. Data are available from the XXX Institutional Data Access / Ethics Committee (contact via XXX) for researchers who meet the criteria for access to confidential data.*<br><br>*The data underlying the results presented in the study are available from (include the name of the third party*	All relevant data are within the manuscript and its Supporting Information files.

and contact information or URL).<br>• This text is appropriate if the data are owned by a third party and authors do not have permission to share the data.<br><br>* typeset	
Additional data availability information:	

Click here to access/download;Manuscript;PM2.5 manu V17 ALL no fig.docx

Association Between PM 2.5 and Effect of Emergency Department Visits for Acute Respiratory Disease, Acute Coronary Syndrome, Acute Heart Failure, and Stroke

Panumas Surit MD[1], Wachira Wongtanasarasin MD[1],

Chiraphat Boonnag MD[2, 3], Borwon Wittayachamnankul MD, PhD[1]

[1] Department of Emergency Medicine, Faculty of Medicine, Chiang Mai University

[2] Department of Biochemistry, Faculty of Medicine, Chiang Mai University

[3] Biomedical Informatics Center, Faculty of Medicine, Chiang Mai University, Chiang Mai, Thailand

Corresponding Author: Borwon Wittayachamnankul, MD, PhD

Department of Emergency Medicine, Faculty of Medicine, Chiang Mai University

Chiang Mai 50200, Thailand

Tel: +66-5393-6722; Email: borwon.witt@cmu.ac.th

Abstract

Background and objective: To determine the association of $PM_{2.5}$ and other pollutants effects on Emergency Department (ED) visits, hospitalization, and mortality with acute respiratory disease, acute coronary syndrome (ACS), acute heart failure (AHF), and stroke.

Methods: This study was a retrospective study with daily data collected on ED visits between 2018 and 2019 at Maharaj Nakorn Chiang Mai Hospital. A distributed lag non-linear model and quasi-Poisson model were used to explore the relationship between air quality parameters and ED visits in each disease.

Results: A total of 3,540 ED visits were recorded during study period. The mean daily $PM_{2.5}$ concentration was 89.0±40.2 µg/m^3. We observed associations between $PM_{2.5}$ concentrations and the ED visits due to ACS on the following day (RR = 1.023, 95% confidence interval [CI]: 1.002-1.044) and two days after exposure (RR = 1.026, 95% CI: 1.005-1.047). Also, subgroup analysis revealed the association between $PM_{2.5}$ concentrations and the ED visits due to pneumonia on the current day (RR =1.071, 95% CI: 1.025-1.118) and on the following day after exposure (RR =1.024, 95% CI: 1.003-1.046). $PM_{2.5}$ associated with increased mortality resulted from ACS on lag day 3 (OR = 1.36, 95% CI: 1.08-1.73). For other air pollutions, PM_{10} is associated with increased ED visits due to COPD/asthma and increased hospitalization in AHF. Also, O_3 and NO_2 are associated with increased ICU admissions and mortality in AHF.

Conclusion: Short-term exposure to $PM_{2.5}$ pollution can increase the risk of ED visits in ACS and acute respiratory disease particularly for pneumonia.

Short Title: $PM_{2.5}$ on ED Visits in Respiratory and CV

Keyword: Air pollutants; Environmental health; Emergency Department; Public Health; Pneumonia

Introduction

Air pollution is a major issue worldwide and the largest environmental problem nowadays especially harmful health effects.[1] Outdoor air pollution is a complex mixture of components, affecting human health include airborne particulate matter (PM), pollutants ozone (O_3), nitrogen dioxide (NO_2), and sulfur dioxide (SO_2).[2] The most health-damaging PMs are those with a diameter of less than 10 µm (PM_{10}) and 2.5 µm ($PM_{2.5}$), which can penetrate and lodge deep inside the lungs.[2,3] Especially $PM_{2.5}$ is the principal air pollutant showing the greatest threat to global public health.[2,3] Both short- and long-term exposure to $PM_{2.5}$ has been associated with health impacts in the multi-organs system via many pathways with the role of oxidative stress in PM-mediated effects, systemic vascular dysfunction and cardiovascular modeling with air pollution, and autonomic dysfunction and activation of central nervous system pathways.[3] Exposure to $PM_{2.5}$ also leads to respiratory problems and the development of atherosclerosis which increases the risk for coronary artery disease and cerebrovascular disease.[3] Several studies have demonstrated the effects of $PM_{2.5}$ on increased morbidity and mortality, emergency visit and hospitalization for acute respiratory disease especially pneumonia, chronic obstructive pulmonary disease (COPD) and asthma, acute coronary syndrome (ACS), acute heart failure (AHF), and stroke.[2–8]

According to the World Health Organization (WHO), global assessment of ambient air pollution exposure and the resulting burden of disease in 2016, Eastern Mediterranean, South-East Asian (SEA), and Western Pacific Regions had some of the highest exposures to air pollution.[9–11] Thailand is a SEA country with exposure to high annual mean WHO Air Quality Guidelines (AQG).[11,12] The area the highest air pollution concentrations is the northern part of Thailand from January to May. The pollution originates from human activity and wildfires.[12] However, no previous study in SEA mentions the effects of $PM_{2.5}$ toward health impacts in ED visits. Therefore, the primary objective of this study was to determine

the association between the increase of fine particular matter ($PM_{2.5}$) including other pollutants and its effect on acute respiratory disease, ACS, AHF, and stroke in the ED visits, ED disposition and in-hospital mortality.

Material and Methods

Data Collection

We conducted a time series analysis between April 2018 and March 2019 at Maharaj Nakorn Chiang Mai Hospital which represents a tertiary-care, university hospital. Inclusion criteria were patients who visited ED with age more than 18 years old and have the current address in Chiang Mai. The exclusion criteria were patients who referred from other hospitals and trauma patients. The study protocol was approved by the Research Ethics Committee, Faculty of Medicine, Chiang Mai University (Permit No. EXEMPTION-6698/2562).

From the electronic medical record (EMR), we extracted patient data with the principal diagnosis of acute respiratory disease, ACS, AHF, and stroke, using the International Classification of Diseases (ICD) 10th revision codes of J00-J99, I20-I25, I50, and I60-I69, respectively. The principal diagnosis was recorded in EMR by physicians. Data collection derived from the EMR included age, gender, visit date, diagnosis, emergency department disposition, and in-hospital mortality.

Daily average concentration and hourly real-time air pollution data for a concentration of $PM_{2.5}$, PM_{10}, ozone (O_3), nitrogen dioxide (NO_2), and sulfur dioxide (SO_2) were collected from the Smoke Haze Integrated Research Unit (SHIRU) and the Air Pollution in Chiang Mai: Real-time Air Quality Index Visual Map website (https://aqicn.org/map/chiang-mai/). There are 12 stations on the map in the Mueang Chiang Mai District of Chiang Mai Province in Thailand, managed by The World Air Quality Index project team. WHO Air Quality Guideline Standard (AQCs) recommend aiming for annual mean $PM_{2.5}$ concentration values of ≤ 10 µg/m^3 and 24-hour mean concentration values of ≤ 25 µg/m^3; the Thai Air Quality

Standard recommends 24-hour average $PM_{2.5}$ concentrations of $\leq 50\ \mu g/m^3$. For other air pollutants, the Thai Air Quality Standard recommends 24-hour average PM_{10} concentrations of $\leq 120\ \mu g/m^3$, 8-hour average O_3 concentrations of $\leq 140\ \mu g/m^3$, 1-hour average NO_2 concentrations of $\leq 320\ \mu g/m^{3,}$ and 1-hour average SO_2 concentrations of $\leq 780\ \mu g/m^3$. The outcome of this study has measured the effects of air pollutions on ED visits, hospitalization, Intensive Care Unit (ICU) admissions, and mortality on acute respiratory disease, ACS, AHF, and stroke in the ED.

In this analysis, results were summarized as medians and interquartile ranges for non-normally distributed variables, and frequency and percentage for categorical variables. The primary exposure variable was $PM_{2.5}$, considering the nonlinear exposure-lag-response relationship between exposure to air pollution and health effects, an additional dimension, temporal dependency of exposure and outcome, was required to characterize and control the model.[13–16] Distributed lag non-linear model (DLNM) is a model that the relationship between air quality parameters and ED visits is described in both the usual predictor and the additional dimension of time lags. The model is defined by the following formula:

$$\log\big(E(Y_t)\big) = \alpha + ns(RH_t, 3) + ns(Temp, 3) + ns(AP_t, 3) + \beta_0 X_t + \cdots + \beta_q X_{t-q} + \varepsilon_t$$

Where $E(Y_t)$ is the expected number of ED visits on day t and α is the model intercept. X_{t-q} represented the focused air pollutants ($PM_{2.5}$ or PM_{10}) concentration q days before ED visit. The model was adjusted for other environmental confounding variables using a natural cubic spline with 3 df for relative humidity (RH_t), temperature (Temp), and other air pollutants (AP_t; PM_{10} (or $PM_{2.5}$), O_3, and NO_2). We investigated the lag structure of $PM_{2.5}$ effects on ED visits using a polynomial function with 7 days from lag 0 to lag 6. The outcome of the DLNM was the count of ED visits in each disease, and the quasi-Poisson model was used. Lastly, a Multivariate logistic regression model was employed to explore independent air quality (PM_{10}, $PM_{2.5}$, O_3, and NO_2) predictors of hospital admission, critical

care unit, and in-hospital death. All statistical analyses were performed using R (version 4.0.0). Two-tailed $p < 0.05$ was considered statistically significant.

Results

A total of 3,540 ED visits were recorded for acute respiratory diseases, ACS, AHF, and stroke, from April 2018 to March 2019, with respiratory disease accounting for the largest proportion (55.7%). Most patients were more than 65 years old (46.3%) and were male (51.5%). The highest percentage of daily ED visits for cause-specific respiratory diseases were Upper Respiratory Tract Infections (URTI) (28.5%), followed by Pneumonia (27.3%), Chronic Obstructive Pulmonary Disease (COPD), and Asthma (26%), respectively. 1,664 out of 3,540 (47%) were admitted to the hospital and 16.6% were admitted to the ICU. Patients diagnosed with ACS had the highest admission rate (80.6%). Moreover, the total in-hospital death was 9.2%, mostly due to respiratory diseases. **Table 1** describes the characteristics of patients presented at the ED during the study period. The overall median daily $PM_{2.5}$ concentration was 75.0 $\mu g/m^3$ (Interquartile range; IQR 63-102 $\mu g/m^3$) and mean daily concentration was 89.0 ± 40.2 $\mu g/m^3$ above from Thai Air Quality Standard recommendations. The median daily concentrations of PM_{10}, O_3, NO_2, and SO_2 were 39 $\mu g/m^3$ (IQR 32-53 $\mu g/m^3$), 19 $\mu g/m^3$ (IQR 15-24 $\mu g/m^3$), 8 $\mu g/m^3$ (IQR 6-12 $\mu g/m^3$) and 0 $\mu g/m^3$, respectively, as shown in **Figure 1 and Supplementary Table 1**. Concentrations of all air pollutants were highest during April of both years (2018 and 2019), as shown in **Supplementary Figure 2**.

Figure 2 shows the association between the adjusted lag-effect of $PM_{2.5} > 50$ $\mu g/m^3$ on ED visits to acute respiratory disease, ACS, AHF, and stroke. We observed statistically significant associations between the ED visits due to ACS and $PM_{2.5}$ concentrations on the following day (lag day 1) (RR = 1.023; 95% CI: 1.002-1.044), two days after exposure (lag day 2) (RR = 1.026; 95% CI: 1.005-1.047) and returned to normal three days after exposure to

$PM_{2.5}$ (lag day 3). No statistically significant associations were found between $PM_{2.5}$ and ED visits due to acute respiratory disease, AHF, and stroke at any lag day.

Analysis of the effect of $PM_{2.5}$ on cause-specific respiratory diseases, including URTI, Pneumonia, COPD, and Asthma **(Figure 3)** found statistically significant associations in ED visits of pneumonia on the current day (lag day 0) (RR = 1.071; 95% CI: 1.025-1.118), lag day 1 (RR = 1.024; 95% CI: 1.003-1.046) then reverted to normal levels at two days after exposure (lag day 2), with the effects rebounding four days after exposure (lag day 4) (RR = 0.970; 95% CI: 0.948-0.993) and five days after exposure (lag day 5) (RR = 0.979; 95% CI: 0.961-0.998). The largest effect was observed on the current day (lag day 0). Moreover, we investigated the effects of other air pollutions, such as PM_{10}, O_3, and NO_2, on ED visits and hospitalizations. Above-normal limits of PM_{10} (>120 μg/m^3) were associated with increased ED visits due to COPD and asthma on lag day three (RR = 1.159; 95% CI: 1.007-1.1335) and lag day four (RR=1.138; 95% CI: 1.009-1.284) **(Supplementary Figure 3).** For O_3 and NO_2, no conclusions could be drawn because their values did not exceed the standard references across the study period.

Figure 4 demonstrates the association between air pollutants and hospitalization for specific diseases at any lag day. We observed statistically significant associations between the hospitalization and AHF with every increase of 10 μg/m^3 in PM_{10} on lag day two (OR = 1.32; 95% CI: 1.04-1.67). However, the statistical results show no significant associations between air pollutants and other diseases. Additionally, this study found statistically significant air pollutants increase the risk effect of ICU admissions and mortality at any lag day as shown in **Supplementary Figures 4 and 5**. Every 10 μg/m^3 increase of NO_2 on the previous two days was associated with an increase in ICU admission for AHF (OR = 1.13; 95% CI: 1.02-1.26). $PM_{2.5}$ was associated with increased ACS mortality on three days after exposure (OR = 1.36; 95% CI: 1.08-1.73). Furthermore, O_3 was associated with increased

AHF mortality on the current day (OR = 1.16; 95% CI: 1.04-1.29), the following day (OR=1.13; 95% CI: 1.04-1.23) and the next two days after exposure (OR = 1.17; 95% CI: 1.05-1.30).

Discussion

This study found that $PM_{2.5}$ concentrations were associated with the number of ED visits due to ACS on the following day and two days after exposure (lag days 1 and 2), and pneumonia on the current day and following day after exposure. Consistent with previous studies, ED visits due to acute respiratory diseases were associated with an increased $PM_{2.5}$ concentration on the same day.[4,6,17–19] Interestingly, no previous studies were found that examined the effects of $PM_{2.5}$ on ED visits in the aforementioned group of diseases conducted in SEA. The previous study did not mention how $PM_{2.5}$ affected ED visits but investigated the effects of $PM_{2.5}$ on daily mortality and hospitalization in cardiovascular and respiratory disease, has found that $PM_{2.5}$ effects in COPD and community-acquired pneumonia.[20] Consistent with the previous study, $PM_{2.5}$ is associated with increased mortality of ACS patients three days after exposure.[18] Additionally, we found that the effect of $PM_{2.5}$ pollution on hospitalization of respiratory disease was significantly greater in males than females and patients ages greater than 65 years. Compared with previous studies, the subgroup analysis results suggested that elderly people might be more susceptible to $PM_{2.5}$ exposure and may have a weaker immune system and a higher prevalence of chronic respiratory diseases.[6,20]

$PM_{2.5}$ was hypothesized to have the most effects on the respiratory and cardiovascular systems: oxidative stress in PM-mediated effects directly affect the respiratory system through inhaled $PM_{2.5}$ depositing deep within pulmonary tissues interacting with local cells and modifying endogenous structures, consequently leading to local inflammatory response. Also, PM-mediated effects influencing the development of atherosclerosis, resulting in an increased risk for coronary artery disease.[2–4] The results of this study indicate that the short-

term effect of ambient $PM_{2.5}$ exposure is associated with increased daily ED visits with ACS. The number of ACS patients increased following the rising of $PM_{2.5}$ concentration by one to two days. Regarding AHF and stroke, and increased $PM_{2.5}$ was not found to affect the number of ED visits in any lag days. Previous studies found that most $PM_{2.5}$ effects were a delay, ranging from seven days or more after exposure to $PM_{2.5}$; moreover, stroke was found to be associated with long exposure to air pollutions.[5,8,21,22]

Other air pollutants also demonstrated some effects on ED patients. Interestingly, PM_{10} is another significant air pollutant that can precipitate emergency illnesses especially cardiovascular and respiratory diseases; however, studies that have explored the effect of PM_{10} concentration and specific diseases are limited.[3,23] Also, our study found that PM_{10} was associated with an increased risk of ED visits due to COPD and asthma. Furthermore, our study revealed the association between PM_{10} concentrations and an increased risk of hospitalization due to AHF. Correlated with the previous study, PM_{10} concentrations between 50-200 $\mu g/m^3$ were an isolated risk factor for hospitalization in AHF patients in Saharan desert dust.[24] Also, NO_2 and O_3 also took part in the pathogenesis of inflammation, oxidative stress, and autonomic abnormality which consequently resulted in an increased risk of unfavorable outcomes in AHF patients.[3,25,26]

This is the first study in SEA studying the effects of $PM_{2.5}$ and PM_{10} on ED visits in a place where the daily $PM_{2.5}$ and PM_{10} concentrations were higher than the standard level (WHO AQGs and Thailand Air Quality Standard). Also, this study demonstrated that the possible health effects of exposure to air pollution, especially effects of $PM_{2.5}$ on cardiovascular and respiratory systems as mentioned. This is of importance for both healthcare providers and public health authorities and could contribute to health promotion for the general public by increasing awareness of the effects of air pollution and preventative measures. Also, to increase government awareness on the gravity of air pollution issues to be

aware of harmfulness and health hazards to the populations in this area. Finally, to help guide systems planning for health care professionals especially those in the emergency services to prepare for increases in ACS and respiratory disease visits to the ED in seasons where $PM_{2.5}$ concentrations are abnormally high, especially, during winter (December to February), and peak again in forest fire season (April) **(Supplementary Figure 1)**. Also, a well-prepared protocol during the season which has a high level of $PM_{2.5}$ is warranted to ensure both patients and healthcare personnel that ED and hospital are administering this issue.

Some limitations of our study should be mentioned. First, it is a single-center study and only conducted in one district; thus, the sample size studied in subgroup analyses was small. Additionally, patients referred from other hospitals were excluded from the study, and as this was conducted in a tertiary teaching hospital, the admission criteria were strict, which may influence the number of admissions in disease subgroup analyses. Second, patient data obtained from the principal diagnosis made in the ED according to ICD-10 did not include disease severity, risk factors, and initial management. Furthermore, our data explored the effects of short-term exposure. Finally, we did not collect data on preventative measures taken by the sample population to lessen $PM_{2.5}$ exposure, such as using home air purifiers and wearing face masks.

Conclusion

Our study provides evidence that short-term $PM_{2.5}$ pollution exposure can increase the risk of ED visits in ACS and respiratory disease, particularly for pneumonia.

Acknowledgment

The authors would like to thank the Faculty of Medicine, Chiang Mai University for funding this research and the Smoke Haze Integrated Research Unit (SHIRU) for supporting the air pollution data. Also, our thanks goes to Miss Rudklao Sairai and colleagues, the Research Unit of Department of Emergency Medicine, Chiang Mai University for providing support to this study. We appreciate Dr. Nattikarn Atthapreyangkul for English language editing and Dr. Phichayut Phinyo for methodology and statistical advice. Finally, we would like to thank the Medical Records and Statistics Section of Maharaj Nakorn Chiang Mai Hospital for providing the data of patients in this study.

Conflict of Interest

None.

Ethics Approval Statement

This research was approved by Research Ethics Committee, Faculty of Medicine, Chiang Mai University (EXEMPTION-6698/2562)

Funding Information

Faculty of Medicine, Chiang Mai University funded this research (Fund No. 089-2563).

References

1. Landrigan PJ, Fuller R, Acosta NJR, Adeyi O, Arnold R, Basu N (Nil), et al. The Lancet Commission on pollution and health. The Lancet. 2018 Feb 3;391(10119):462–512.

2. Newby DE, Mannucci PM, Tell GS, Baccarelli AA, Brook RD, Donaldson K, et al. Expert position paper on air pollution and cardiovascular disease. European Heart Journal. 2015 Jan 7;36(2):83–93.

3. Rajagopalan S, Al-Kindi SG, Brook RD. Air Pollution and Cardiovascular Disease: JACC State-of-the-Art Review. Journal of the American College of Cardiology. 2018 Oct 23;72(17):2054–70.

4. Haikerwal A, Akram M, Del Monaco A, Smith K, Sim MR, Meyer M, et al. Impact of Fine Particulate Matter ($PM_{2.5}$) Exposure During Wildfires on Cardiovascular Health Outcomes. Journal of the American Heart Association. 2015 Jul 17;4(7):1–10.

5. Shah AS, Langrish JP, Nair H, McAllister DA, Hunter AL, Donaldson K, et al. Global association of air pollution and heart failure: a systematic review and meta-analysis. The Lancet. 2013 Sep 21;382(9897):1039–48.

6. Xu Q, Li X, Wang S, Wang C, Huang F, Gao Q, et al. Fine Particulate Air Pollution and Hospital Emergency Room Visits for Respiratory Disease in Urban Areas in Beijing, China, in 2013. PLOS ONE. 2016;11(4):e0153099.

7. Jo YS, Lim MN, Han YJ, Kim WJ. Epidemiological study of $PM_{2.5}$ and risk of COPD-related hospital visits in association with particle constituents in Chuncheon, Korea [Internet]. International Journal of Chronic Obstructive Pulmonary Disease. 2018 [cited 2019 Mar 31]. Available from: https://www.dovepress.com/epidemiological-study-of-pm25-and-risk-of-copd-related-hospital-visits-peer-reviewed-fulltext-article-COPD

8. Shah ASV, Lee KK, McAllister DA, Hunter A, Nair H, Whiteley W, et al. Short term exposure to air pollution and stroke: systematic review and meta-analysis. BMJ. 2015 Mar 24;350:h1295.

9. Ambient (outdoor) air quality and health [Internet]. [cited 2019 Apr 1]. Available from: https://www.who.int/news-room/fact-sheets/detail/ambient-(outdoor)-air-quality-and-health

10. WHO | Ambient air pollution: Health impacts [Internet]. WHO. [cited 2019 Apr 1]. Available from: http://www.who.int/airpollution/ambient/health-impacts/en/

11. WHO | Ambient air pollution: A global assessment of exposure and burden of disease [Internet]. WHO. [cited 2019 Apr 1]. Available from: http://www.who.int/phe/publications/air-pollution-global-assessment/en/

12. Pollution Control Department, Ministry of Natural Resources and Environment. Thailand Pollution Report 2018_Thai [Internet]. S. MONGKOL PRESS LTD., PART.; 2562. 19–33 p. Available from: http://www.pcd.go.th/file/Thailand%20Pollution%20Report%202018_Thai.pdf

13. Armstrong B. Models for the Relationship Between Ambient Temperature and Daily Mortality. Epidemiology. 2006 Nov;17(6):624–31.

14. Schwartz J. The Distributed Lag between Air Pollution and Daily Deaths. Epidemiology. 2000 May;11(3):320–6.

15. Chen F, Deng Z, Deng Y, Qiao Z, Lan L, Meng Q, et al. Attributable risk of ambient PM10 on daily mortality and years of life lost in Chengdu, China. Science of The Total Environment. 2017 Mar;581–582:426–33.

16. Guo Y, Ma Y, Zhang Y, Huang S, Wu Y, Yu S, et al. Time series analysis of ambient air pollution effects on daily mortality. Environ Sci Pollut Res. 2017 Sep;24(25):20261–72.

17. Li T, Hu R, Chen Z, Li Q, Huang S, Zhu Z, et al. Fine particulate matter (PM2.5): The culprit for chronic lung diseases in China. Chronic Diseases and Translational Medicine. 2018 Sep 1;4(3):176–86.

18. Meng X, Zhang Y, Yang K-Q, Yang Y-K, Zhou X-L. Potential Harmful Effects of PM2.5 on Occurrence and Progression of Acute Coronary Syndrome: Epidemiology, Mechanisms, and Prevention Measures. IJERPH. 2016 Jul 25;13(8):748.

19. Pope CA, Muhlestein JB, May HT, Renlund DG, Anderson JL, Horne BD. Ischemic Heart Disease Events Triggered by Short-Term Exposure to Fine Particulate Air Pollution. Circulation. 2006 Dec 5;114(23):2443–8.

20. Pothirat C, Chaiwong W, Liwsrisakun C, Bumroongkit C, Deesomchok A, Theerakittikul T, et al. Acute effects of air pollutants on daily mortality and hospitalizations due to cardiovascular and respiratory diseases. 2019. 2019;11(7):3070–83.

21. Li M, Wu Y, Tian Y-H, Cao Y-Y, Song J, Huang Z, et al. Association Between PM2.5 and Daily Hospital Admissions for Heart Failure: A Time-Series Analysis in Beijing. International Journal of Environmental Research and Public Health. 2018 Oct;15(10):2217.

22. Tian Y, Xiang X, Wu Y, Cao Y, Song J, Sun K, et al. Fine Particulate Air Pollution and First Hospital Admissions for Ischemic Stroke in Beijing, China. Sci Rep. 2017 20;7(1):3897.

23. Feng W, Li H, Wang S, Van Halm-Lutterodt N, An J, Liu Y, et al. Short-term PM10 and emergency department admissions for selective cardiovascular and respiratory diseases in Beijing, China. Science of The Total Environment. 2019 Mar 20;657:213–21.

24. Domínguez-Rodríguez A, Baez-Ferrer N, Rodríguez S, Abreu-González P, González-Colaço Harmand M, Amarnani-Amarnani V, et al. Impact of exposure of emergency patients with acute heart failure to atmospheric Saharan desert dust. Emergencias. 2019 Jun;31(3):161–6.

25. Srebot V, Gianicolo EA, Rainaldi G, Trivella MG, Sicari R. Ozone and cardiovascular injury. Cardiovascular Ultrasound. 2009 Jun 24;7(1):1–8.

26. Chiusolo Monica, Cadum Ennio, Stafoggia Massimo, Galassi Claudia, Berti Giovanna, Faustini Annunziata, et al. Short-Term Effects of Nitrogen Dioxide on Mortality and Susceptibility Factors in 10 Italian Cities: The EpiAir Study. Environmental Health Perspectives. 2011 Sep 1;119(9):1233–8.

Table 1. Descriptive Summary for the Study Period (April 2018 to March 2019), Emergency Department Visits, ICU Admission and In-Hospital Deaths for Acute Respiratory Diseases, Acute Coronary Syndrome, Acute Heart Failure and Stroke

Characteristics	Total	Acute Respiratory Disease (ICD10 code: J00-J99)	Acute Coronary Syndrome (ICD10 code: I20-I25)	Acute Heart Failure (ICD10 code: I50)	Stroke (ICD10 code: I60-69)
	N (%)	N (%)	N (%)	N (%)	N (%)
ED visit	3540 (100)	1973 (55.7)	470 (13.3)	365 (10.3)	732 (20.7)
Median age – year (IQR)	63 (29)	59 (44)	65 (15.8)	69 (20)	63 (21)
Age (year) *					
18-34	637 (18.0)	596 (30.2)	1 (0.2)	8 (2.2)	32 (4.4)
35-44	207 (5.8)	135 (6.8)	18 (3.8)	14 (3.8)	40 (5.5)
45-54	349 (9.9)	141 (7.2)	59 (12.6)	35 (9.6)	114 (15.6)
55-64	705 (20.0)	291 (14.8)	140 (29.8)	67 (18.4)	207 (28.3)
≥ 65	1638 (46.3)	810 (41.0)	252 (53.6)	241 (66.0)	335 (45.8)
Gender					
Male	1824 (51.5)	988 (50.1)	293 (62.3)	158 (43.3)	385 (52.6)
Female	1716 (48.5)	985 (49.9)	177 (37.7)	207 (56.7)	347 (47.4)
Disposition					
Discharge	1876 (53.0)	1414 (71.7)	91 (19.4)	156 (42.7)	215 (29.4)
Total Admission	1664 (47.0)	559 (28.3)	379 (80.6)	209 (57.3)	517 (70.6)
ICU Admission	276 (16.6)	22 (3.9)	218 (57.5)	20 (9.6)	16 (3.09)
In-hospital death	153 (9.2)	75 (13.4)	27 (7.1)	19 (9.1)	32 (6.2)

** Note: Age (N/A = 4; ICD10 I60-I69 = 4*

Figure legend

Figure 1. Box Plot of Daily Ambient Data for the Study Period (April 2018-March 2019)

Figure 2. Relative Risk of the Adjusted Lag-Effect between $PM_{2.5}$ and ED Visits of Respiratory Disease, Acute Coronary Syndrome, Acute Heart Failure and Stroke (Reference $PM_{2.5}$ = 50 μg/m^3)

Figure 3. Relative Risk of the Adjusted Lag-Effect between $PM_{2.5}$ and ED Visits of Cause-Specific of Respiratory Disease; URTI, Pneumonia, COPD and Asthma

(Reference $PM_{2.5}$ = 50 μg/m^3)

Figure 4. Excess Risk (95% Confident Intervals) of Association between Air Pollutants (PM2.5, PM10, O3 and NO2) and Hospitalization for Serious Specific Disease at Lag Day 0-5

Figure 1

Click here to access/download;Figure;fig1.png

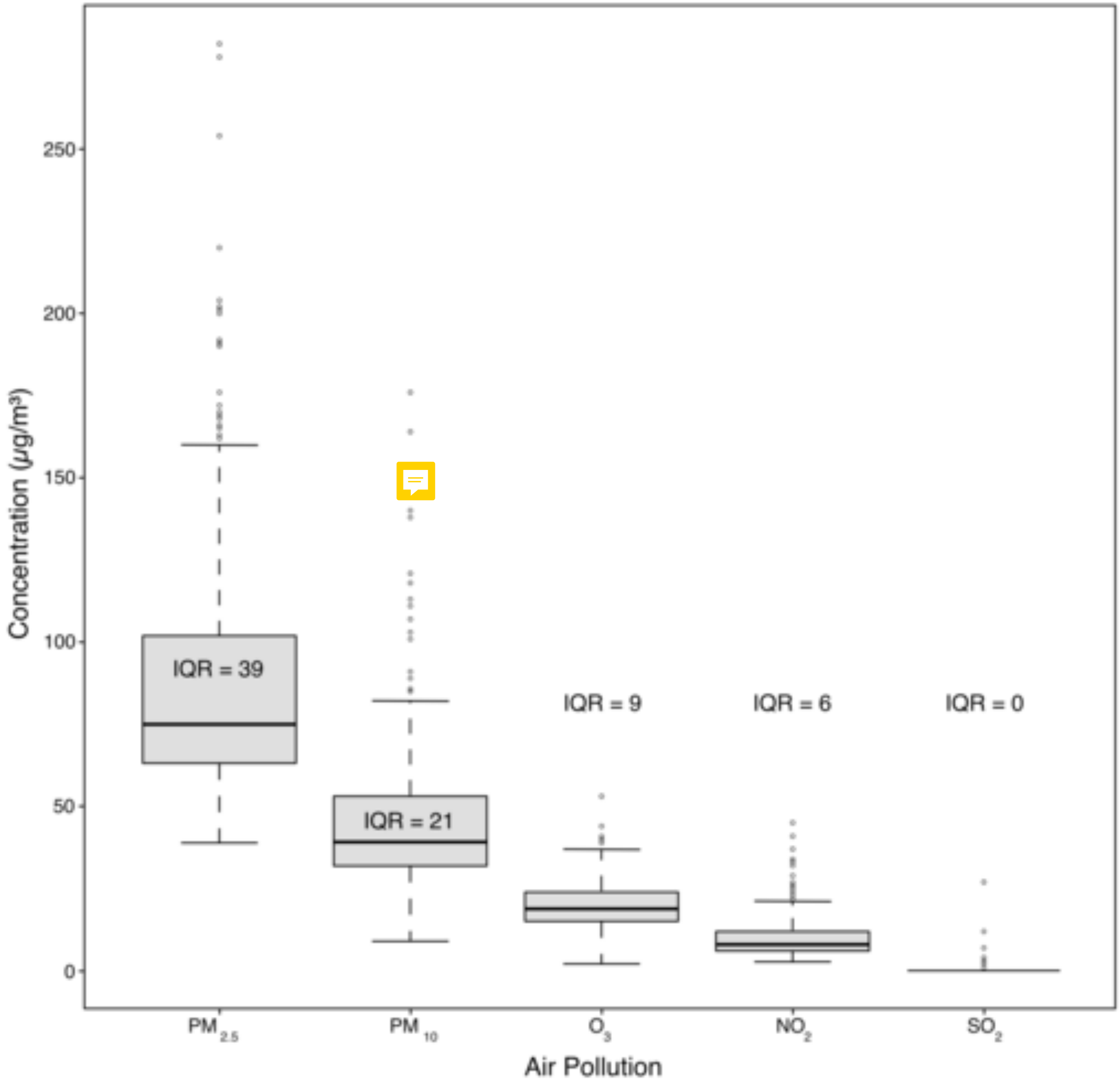

Figure 2

Click here to access/download;Figure;fig 2.png

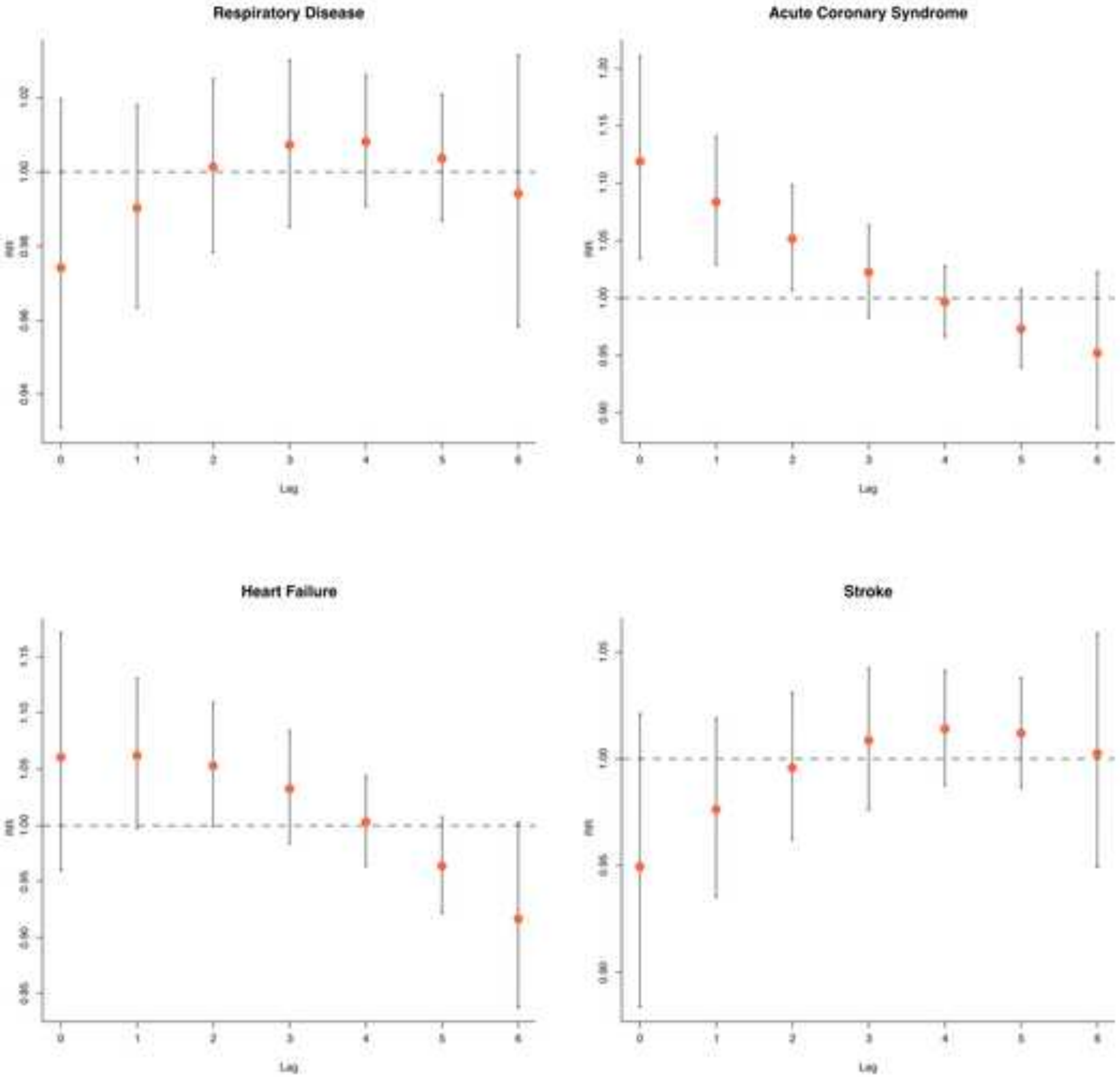

Click here to access/download;Figure;fig 3.png

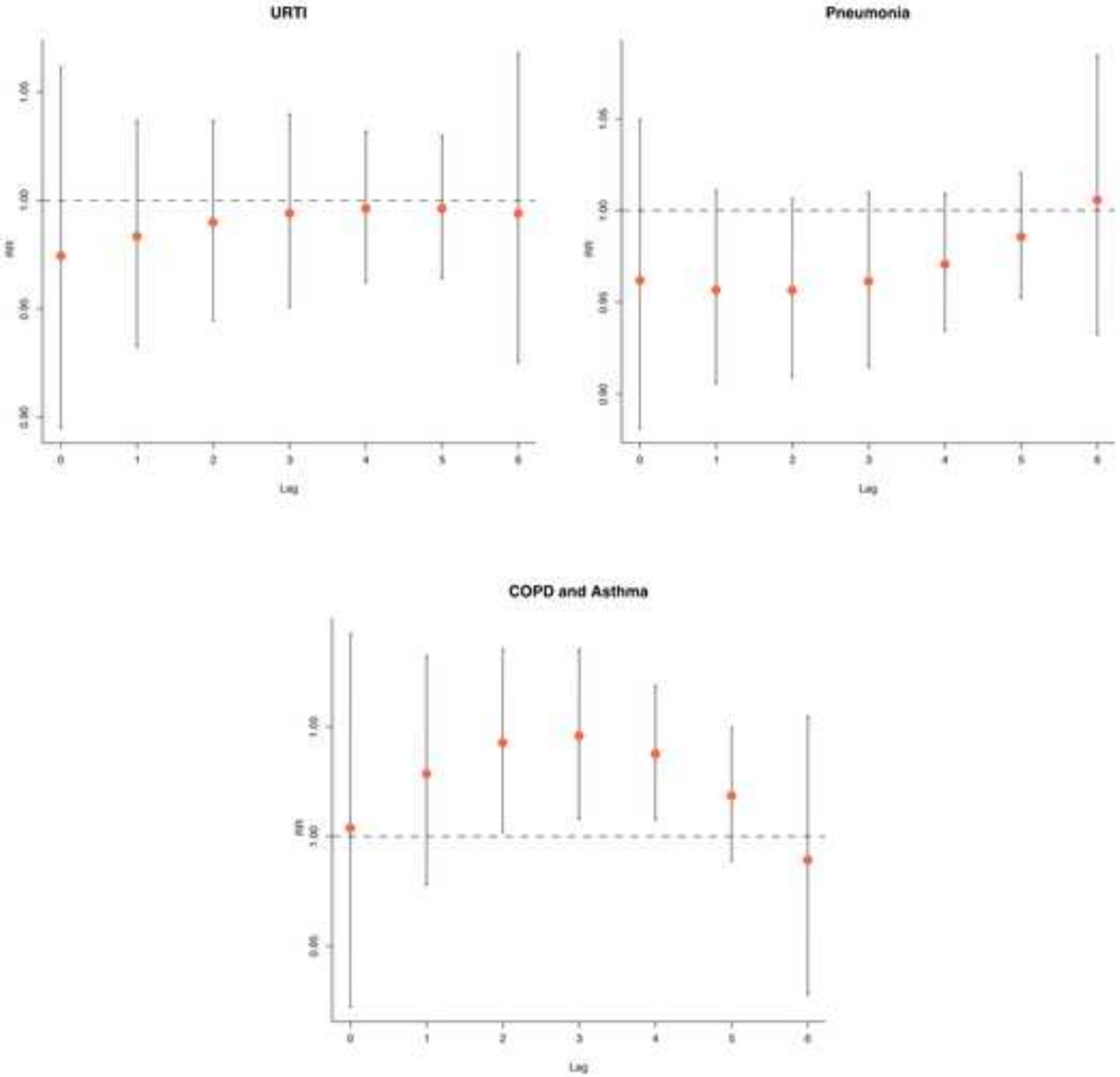

Figure 4

Click here to access/download;Figure;fig 4.png

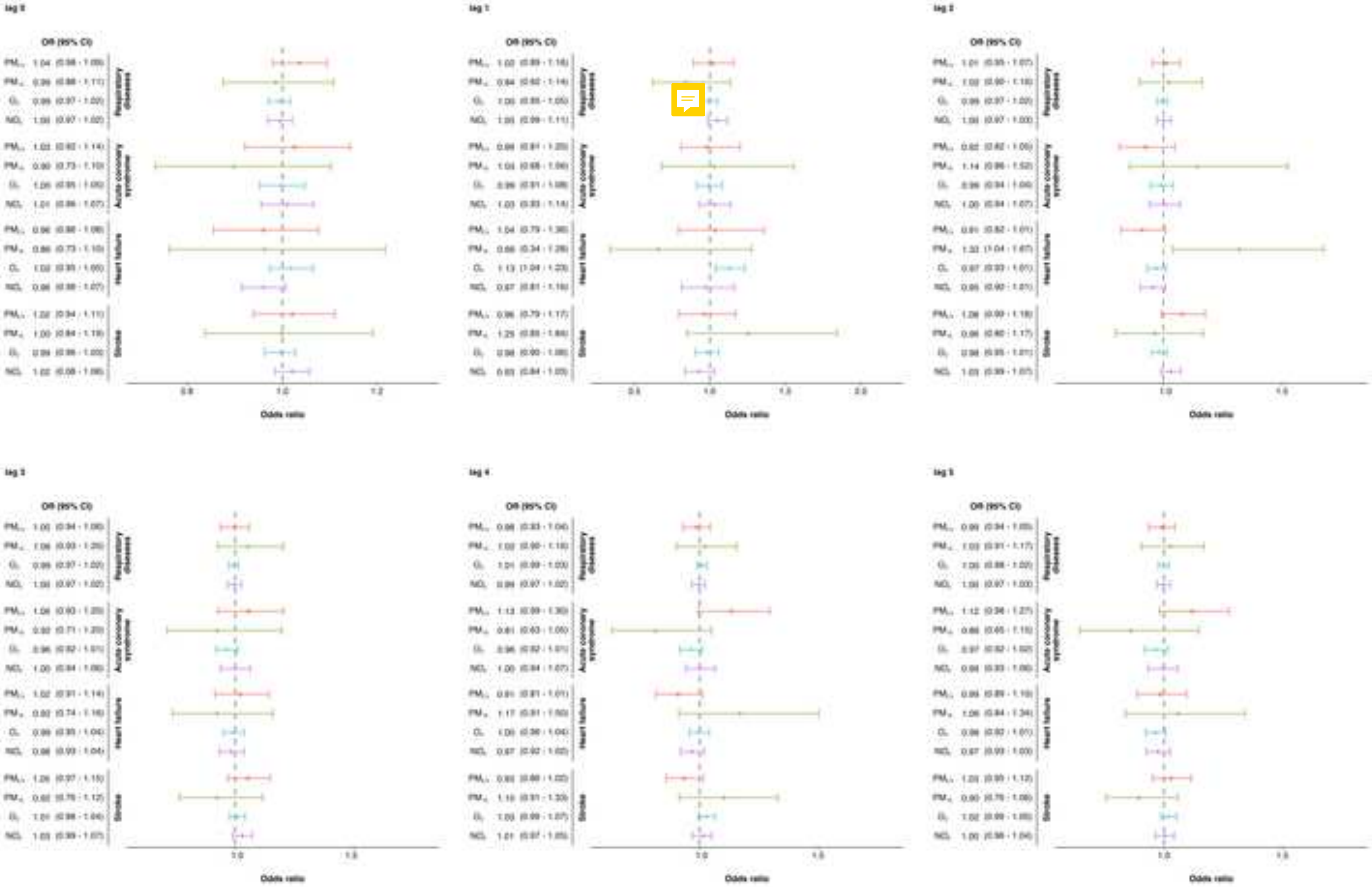

Supplement: Attachment — Submitted filename: PONE-D-22-17550_reviewer.pdf [file pone.0294107.s004.pdf]
