# Supplementary material for: Association between air quality index and effects on emergency department visits for acute respiratory and cardiovascular diseases
Source: PLoS One. 2023 Nov 16;18(11):e0294107. doi: 10.1371/journal.pone.0294107 (PMC10653395; doi:10.1371/journal.pone.0294107)
Supplement: Attachment — Submitted filename: 20220812_Reviewer_MJ Ting.doc [file pone.0294107.s003.doc]

**Dear Dr. Oyelola A. Adegboye,**

Thank you for inviting me to review this manuscript. Overall this study is well done and interesting. The paper could be accepted for publication into PLOS ONE after minor revisions.

**Comments to the Authors:**

**Title P.1**

The title of your study is “association between PM 2.5 and effect of emergency department visits for acute respiratory disease, acute coronary syndrome, acute heart failure, and stroke.” However, the authors didn’t only focus on PM2.5, but also on other air pollutants, including PM10, O3, NO2, SO2. A suitable study title should contain the critical keywords and predict the content of the research. Therefore, the keyword "PM 2.5" may be revised as "air pollutants."

**Material and Methods P. 4**

Your study investigated short-term air pollutant exposures and emergency department visits for acute respiratory diseases and cardiovascular diseases between 2018 and 2019. In general, the study periods of epidemiologic studies regarding the relationship between short-term exposure to particulate matter and diseases are more than one year or at least two years. It would be better if the time series analysis from 2018 to 2020 could be conducted.

**Material and Methods P. 4**
The sampling of this study is not representative of the exposure-outcome distributions in the overall population. Selection bias could have occurred when investigators use only one hospital data.

**Material and Methods P. 4**

The inclusion criteria of the study subjects are not clear. How did you define daily counts of emergency department visits for acute respiratory diseases and cardiovascular diseases? by ICD10 or ICD10 plus some kind of treatments from hospital electronic medical records?

**Material and Methods P. 5**
The analyses consider several important confounding variables, including age, gender, and season.

**Discussion P.9**

Although unmeasured confounding remained in this study, you provide important new evidence that short-term PM2.5, PM10 and other air pollutant exposures is significantly related to emergency department visits for acute respiratory diseases and cardiovascular diseases. Further research might be conducted to investigate the associations of long-term exposure to fine particulate matter and air pollutants with healthcare utilization.

**Discussion P.10**

According to previous studies, Chiang Mai has been facing severe problem of haze pollution over the past years. Did emergency department visits to treat respiratory diseases and cardiovascular diseases increase during haze episodes in your study? Had Maharaj Nakorn Chiang Mai Hospital been prepared to meet the surge in demand for medical treatment? It could be one of the confounding factors in your study and may produce spurious or distorted associations between exposure to air pollutants and increased emergency department visits. So, the authors may add methods to decrease the impact of confounding variables in the study design. This requires clarification.

**Discussion P.10**

Chiang Mai is surrounded by the mountain ranges of the Thai highlands and has a tropical savanna climate with hot, wet temperatures year-round. Many studies have shown the meteorological factors could impact the PM concentrations, including the dispersion, growth, chemical production, photolysis, and deposition of PM. Besides, the dominant meteorological factors for PM concentrations are closely related to geographical conditions. Also, weather conditions could trigger respiratory symptoms in patients with respiratory diseases. Thus, I suggest that the effects of PM concentrations on multiple meteorological factors, including cloud cover, temperature, wind, humidity, relative humidity, pressure, precipitation, rainfall, radiation and planetary boundary layer height might be included and carefully examined in the further study.

**Conclusion P.10**

The authors should be cautious in interpreting your results. Many studies focusing on PM2.5 and health outcomes may overstate conclusions of their findings.
