# Supplementary material for: Association between air quality index and effects on emergency department visits for acute respiratory and cardiovascular diseases
Source: PLoS One. 2023 Nov 16;18(11):e0294107. doi: 10.1371/journal.pone.0294107 (PMC10653395; doi:10.1371/journal.pone.0294107)
Supplement: Attachment — Submitted filename: P_ONE_cb.docx [file pone.0294107.s005.docx]

**Response to reviewer and editor**

1. Please ensure that your manuscript meets PLOS ONE's style requirements, including those for file naming. The PLOS ONE style templates can be found at 
https://journals.plos.org/plosone/s/file?id=wjVg/PLOSOne_formatting_sample_main_body.pdf and 
https://journals.plos.org/plosone/s/file?id=ba62/PLOSOne_formatting_sample_title_authors_affiliations.pdf

Answer: thank you for suggestion. We edit the revised manuscript to PLOS ONE's style requirements

2. Please provide additional details regarding participant consent. In the ethics statement in the Methods and online submission information, please ensure that you have specified (1) whether consent was informed and (2) what type you obtained (for instance, written or verbal, and if verbal, how it was documented and witnessed). If your study included minors, state whether you obtained consent from parents or guardians. If the need for consent was waived by the ethics committee, please include this information.
If you are reporting a retrospective study of medical records or archived samples, please ensure that you have discussed whether all data were fully anonymized before you accessed them and/or whether the IRB or ethics committee waived the requirement for informed consent. If patients provided informed written consent to have data from their medical records used in research, please include this information.

Answer: We retrospective collected data and the need for consent was waived by the ethics committee. We add this information in the revised manuscript.

3. Thank you for stating the following financial disclosure: 
   "Faculty of Medicine, Chiang Mai University funded this research (Fund No. 089-2563)."

Please state what role the funders took in the study.  If the funders had no role, please state: "The funders had no role in study design, data collection and analysis, decision to publish, or preparation of the manuscript." 
If this statement is not correct you must amend it as needed. 
Please include this amended Role of Funder statement in your cover letter; we will change the online submission form on your behalf.

Answer: Thanks, we change this in revised manuscript.

4. Thank you for stating the following in the Acknowledgments Section of your manuscript: 
  "The authors would like to thank the Faculty of Medicine, Chiang Mai University for funding this research and the Smoke Haze Integrated Research Unit (SHIRU) for supporting the air pollution data. Also, our thanks goes to Miss Rudklao Sairai and colleagues, the Research Unit of Department of Emergency Medicine, Chiang Mai University for providing support to this study. We appreciate Dr. Nattikarn Atthapreyangkul for English language editing and Dr.Phichayut Phinyo for methodology and statistical advice. Finally, we would like to thank the Medical Records and Statistics Section of Maharaj Nakorn Chiang Mai Hospital for providing the data of patients in this study."

We note that you have provided funding information that is not currently declared in your Funding Statement. However, funding information should not appear in the Acknowledgments section or other areas of your manuscript. We will only publish funding information present in the Funding Statement section of the online submission form. 
Please remove any funding-related text from the manuscript and let us know how you would like to update your Funding Statement. Currently, your Funding Statement reads as follows: 
 "Faculty of Medicine, Chiang Mai University funded this research (Fund No. 089-2563)."

Answer: Thanks, we change this in revised manuscript.

5. In your Data Availability statement, you have not specified where the minimal data set underlying the results described in your manuscript can be found. PLOS defines a study's minimal data set as the underlying data used to reach the conclusions drawn in the manuscript and any additional data required to replicate the reported study findings in their entirety. All PLOS journals require that the minimal data set be made fully available. For more information about our data policy, please see http://journals.plos.org/plosone/s/data-availability.

"Upon re-submitting your revised manuscript, please upload your study’s minimal underlying data set as either Supporting Information files or to a stable, public repository and include the relevant URLs, DOIs, or accession numbers within your revised cover letter. For a list of acceptable repositories, please see http://journals.plos.org/plosone/s/data-availability#loc-recommended-repositories. Any potentially identifying patient information must be fully anonymized.

Important: If there are ethical or legal restrictions to sharing your data publicly, please explain these restrictions in detail. Please see our guidelines for more information on what we consider unacceptable restrictions to publicly sharing data: http://journals.plos.org/plosone/s/data-availability#loc-unacceptable-data-access-restrictions. Note that it is not acceptable for the authors to be the sole named individuals responsible for ensuring data access.

Answer: We will update your Data Availability statement to reflect the information you provide in your cover letter.

6. Please upload a copy of Supporting Information Figures 1, 2, 3, 4 and 5 which you refer to in your text on page 10,6,7 .

Answer: Thank you, we just upload this information in revised manuscript

7. Please upload a copy of Supporting Information Table 1 which you refer to in your text on page 6.

Answer: Thank you, we just upload this information in revised manuscript

[Note: HTML markup is below. Please do not edit.]

Reviewers' comments:

Reviewer's Responses to Questions

**Comments to the Author**

1. Is the manuscript technically sound, and do the data support the conclusions?

The manuscript must describe a technically sound piece of scientific research with data that supports the conclusions. Experiments must have been conducted rigorously, with appropriate controls, replication, and sample sizes. The conclusions must be drawn appropriately based on the data presented.

Reviewer #1: Yes

Reviewer #2: Partly

2. Has the statistical analysis been performed appropriately and rigorously?

Reviewer #1: Yes

Reviewer #2: No

3. Have the authors made all data underlying the findings in their manuscript fully available?

The [PLOS Data policy](http://www.plosone.org/static/policies.action#sharing) requires authors to make all data underlying the findings described in their manuscript fully available without restriction, with rare exception (please refer to the Data Availability Statement in the manuscript PDF file). The data should be provided as part of the manuscript or its supporting information, or deposited to a public repository. For example, in addition to summary statistics, the data points behind means, medians and variance measures should be available. If there are restrictions on publicly sharing data—e.g. participant privacy or use of data from a third party—those must be specified.

Reviewer #1: Yes

Reviewer #2: No

4. Is the manuscript presented in an intelligible fashion and written in standard English?

PLOS ONE does not copyedit accepted manuscripts, so the language in submitted articles must be clear, correct, and unambiguous. Any typographical or grammatical errors should be corrected at revision, so please note any specific errors here.

Reviewer #1: Yes

Reviewer #2: No

5. Review Comments to the Author

Please use the space provided to explain your answers to the questions above. You may also include additional comments for the author, including concerns about dual publication, research ethics, or publication ethics. (Please upload your review as an attachment if it exceeds 20,000 characters)

Reviewer #1: Dear Dr. Oyelola A. Adegboye,

Thank you for inviting me to review this manuscript. Overall this study is well done and interesting. The paper could be accepted for publication into PLOS ONE after minor revisions.

Comments to the Authors:

Title P.1
The title of your study is “association between PM 2.5 and effect of emergency department visits for acute respiratory disease, acute coronary syndrome, acute heart failure, and stroke.” However, the authors didn’t only focus on PM2.5, but also on other air pollutants, including PM10, O3, NO2, SO2. A suitable study title should contain the critical keywords and predict the content of the research. Therefore, the keyword "PM 2.5" may be revised as "air pollutants."

Answer: Thanks, we change this in revised manuscript.

Material and Methods P. 4
Your study investigated short-term air pollutant exposures and emergency department visits for acute respiratory diseases and cardiovascular diseases between 2018 and 2019. In general, the study periods of epidemiologic studies regarding the relationship between short-term exposure to particulate matter and diseases are more than one year or at least two years. It would be better if the time series analysis from 2018 to 2020 could be conducted.

Answer: Thank you for your suggestion, however, we have only one year of ER visit data we add this in limitation of revised manuscript.

Material and Methods P. 4
The sampling of this study is not representative of the exposure-outcome distributions in the overall population. Selection bias could have occurred when investigators use only one hospital data.

Answer: Thank you, we already mentioned in discussion.

Material and Methods P. 4
The inclusion criteria of the study subjects are not clear. How did you define daily counts of emergency department visits for acute respiratory diseases and cardiovascular diseases? by ICD10 or ICD10 plus some kind of treatments from hospital electronic medical records?

Answer: Thanks, we already edited this in methods part

Material and Methods P. 5
The analyses consider several important confounding variables, including age, gender, and season.

Answer: Thank you.

Discussion P.9
Although unmeasured confounding remained in this study, you provide important new evidence that short-term PM2.5, PM10 and other air pollutant exposures is significantly related to emergency department visits for acute respiratory diseases and cardiovascular diseases. Further research might be conducted to investigate the associations of long-term exposure to fine particulate matter and air pollutants with healthcare utilization.

- Answer: We add this point in discussion section, thanks for suggestion.

Discussion P.10
According to previous studies, Chiang Mai has been facing severe problem of haze pollution over the past years. Did emergency department visits to treat respiratory diseases and cardiovascular diseases increase during haze episodes in your study? Had Maharaj Nakorn Chiang Mai Hospital been prepared to meet the surge in demand for medical treatment? It could be one of the confounding factors in your study and may produce spurious or distorted associations between exposure to air pollutants and increased emergency department visits. So, the authors may add methods to decrease the impact of confounding variables in the study design. This requires clarification.

- Answer: Our ED has a plan for ED overcrowding, since haze pollution occur this does not impact care process in our ED. However ED overcrowded may not impact the outcome of this research.

Discussion P.10
Chiang Mai is surrounded by the mountain ranges of the Thai highlands and has a tropical savanna climate with hot, wet temperatures year-round. Many studies have shown the meteorological factors could impact the PM concentrations, including the dispersion, growth, chemical production, photolysis, and deposition of PM. Besides, the dominant meteorological factors for PM concentrations are closely related to geographical conditions. Also, weather conditions could trigger respiratory symptoms in patients with respiratory diseases. Thus, I suggest that the effects of PM concentrations on multiple meteorological factors, including cloud cover, temperature, wind, humidity, relative humidity, pressure, precipitation, rainfall, radiation and planetary boundary layer height might be included and carefully examined in the further study.

Answer: Thank you for your suggestion, we add this in limitation of revised manuscript.

Conclusion P.10
The authors should be cautious in interpreting your results. Many studies focusing on PM2.5 and health outcomes may overstate conclusions of their findings.

Reviewer #2: This is an important research study into the acute effects of ambient air pollutants on ED visits for a variety of impotant outcomes. However, there are several limitations that either make me doubt the results and/or formations of the analysis that are unclear and incomplete. Major and minor comments are provided below.

Major Comments:
1. The exposure assessment metrics are not clearly stated in the abstract or in the methods. How were pollutants measured? What is the LOD and how were below LOD values addressed? So2 cannot be 0ug/m3, which makes me concerned about how below LOD values were handled.

Answer: Sorry about the mistakes, we check this with SHIRU and found that this data was AQI not concentration, we have change to AQI not concentration.

2. It is unclear why PM2.5 was only modeled as 50ug/m3 as the reference group. What is the rationnale for this? Why not model a continuous measure? This analysis seems quite incomplete to me.

Answer: 50 (AQI) is the upper limit of good level of AQI, from our reference.

3. PM10 was substantially lower than PM2.5. This practically speaking cannot be possible because PM2.5 is a fraction of PM10. Authors need to explain this oddity. Was PM2.5 and PM10 somehow swapped? This is a major gap in the data that is not addressed by the authors.

Answer: From the mistake that we mention earlier, we check this with SHIRU and found that this data was AQI not concentration, we have change to AQI not concentration.

4. There is a lot of ambiguities that need clearing up, which I believe is largely due to issues with the grammar. For instance, referring to exposure on the following day in the abstract, and repeating it later, makes it sound like exposure effects on the day after ED visit was associated with ED visits. This is inconsistent with how other day lags are talked about in the paper.

Answer: Thank you for your suggestion. We edit our manuscript many points.
5. Overall, there needs to be much more extensive english language editing to fix the clarity of this manuscript.

Answer: In revised version, we edit by English language specialist.

6. Study figures, especially the last two, are of very poor quality and mostly unreadable. These must be more clear for the reader to understand the data.
Answer: Our picture was more than 900 DPI, in our opinion, the publisher was reduce resolution of before send to the reviewer.

Minor Comments:
1. The abstact background is lacking background information.

Answer: we just added this in formation in abstract.

2. the abstract methods need to specify the study area (city, country) and the exposure assessment method

Answer: Thank you, we just added this in formation in abstract.

3. What statistical package (and version number) did authors use for dlnm modeling? Need to specify these

Answer: Thank you, we just added this in formation in abstract.

6. PLOS authors have the option to publish the peer review history of their article ([what does this mean?](https://journals.plos.org/plosone/s/editorial-and-peer-review-process#loc-peer-review-history)). If published, this will include your full peer review and any attached files.


If you choose “no”, your identity will remain anonymous but your review may still be made public.


**Do you want your identity to be public for this peer review?** For information about this choice, including consent withdrawal, please see our [Privacy Policy](https://www.plos.org/privacy-policy).

Reviewer #1: **Yes:** Man-Ju Ting

Reviewer #2: **Yes:** Eric S Coker

[NOTE: If reviewer comments were submitted as an attachment file, they will be attached to this email and accessible via the submission site. Please log into your account, locate the manuscript record, and check for the action link "View Attachments". If this link does not appear, there are no attachment files.]

While revising your submission, please upload your figure files to the Preflight Analysis and Conversion Engine (PACE) digital diagnostic tool, <https://pacev2.apexcovantage.com/>. PACE helps ensure that figures meet PLOS requirements. To use PACE, you must first register as a user. Registration is free. Then, login and navigate to the UPLOAD tab, where you will find detailed instructions on how to use the tool. If you encounter any issues or have any questions when using PACE, please email PLOS at [figures@plos.org](mailto:figures@plos.org). Please note that Supporting Information files do not need this step.
