# Supplementary material for: Association between air quality index and effects on emergency department visits for acute respiratory and cardiovascular diseases
Source: PLoS One. 2023 Nov 16;18(11):e0294107. doi: 10.1371/journal.pone.0294107 (PMC10653395; doi:10.1371/journal.pone.0294107)
Supplement: Attachment — Submitted filename: 20221121 PLOS ONE reviewer_1.doc [file pone.0294107.s006.doc]

**Dear editors and authors,**

Thank you for your thoughtful revisions to the manuscript. Authors have revised some issues of concern that I outlined and have stated the study limitations that may affect the interpretation of the results in a balanced tone. I appreciate the effort the authors put into this work. The article is clearer, and the research question is interesting. However, there are still several points should be addressed before this manuscript can be suitable for publication.

1. Editing for the English language is still required. Please revise and perfect your manuscript by a professional language polishing service or a native English speaker, and attach the language editing certificate when resubmitting the manuscript.

**2. Title, page 1, line 1-2.**

The title of your manuscript has changed to “Association Between Air Quality Index and Effect of Emergency Department Visits for Acute Respiratory Disease, Acute Coronary Syndrome, Acute Heart Failure, and Stroke.”  It's probably not written in standard English, so the authors may discuss with a native English speaker about that.

**3. Abstract, page 2, line 12.**

It is not appropriate to add website address in the abstract section. “(<https://aqicn.org/map/chiang-mai/>)” could be eliminated and should be included in the reference section.
