# Supplementary material for: Association between air quality index and effects on emergency department visits for acute respiratory and cardiovascular diseases
Source: PLoS One. 2023 Nov 16;18(11):e0294107. doi: 10.1371/journal.pone.0294107 (PMC10653395; doi:10.1371/journal.pone.0294107)
Supplement: Attachment — Submitted filename: Response BW edit 02.16.23 .docx [file pone.0294107.s007.docx]

**Response to Reviewer**

**Reviewer #1:** Dear editors and authors,

Thank you for your thoughtful revisions to the manuscript. Authors have revised some issues of concern that I outlined and have stated the study limitations that may affect the interpretation of the results in a balanced tone. I appreciate the effort the authors put into this work. The article is clearer, and the research question is interesting. However, there are still several points should be addressed before this manuscript can be suitable for publication.

1. Editing for the English language is still required. Please revise and perfect your manuscript by a professional language polishing service or a native English speaker and attach the language editing certificate when resubmitting the manuscript.

**Answer:** Thank you for your feedback. We have taken your suggestion seriously and have had the manuscript professionally edited by a native English speaker. We have attached the edited manuscript along with the language editing certificate to this resubmission. We hope that you find the language of the revised manuscript to be improved and suitable for publication.

2. Title, page 1, line 1-2.

The title of your manuscript has changed to “Association Between Air Quality Index and Effect of Emergency Department Visits for Acute Respiratory Disease, Acute Coronary Syndrome, Acute Heart Failure, and Stroke.” It's probably not written in standard English, so the authors may discuss with a native English speaker about that.

**Answer:** Thank you for bringing this to our attention. We have revised the title: "Association Between Air Quality Index and Effects on Emergency Department Visits for Acute Respiratory and Cardiovascular Diseases." We believe that this new title accurately reflects the content of the manuscript and is written in standard English.

3. Abstract, page 2, line 12.

It is not appropriate to add website address in the abstract section. “(https://aqicn.org/map/chiang-mai/)” could be eliminated and should be included in the reference section.

**Answer:** Thank you for pointing out this error in our abstract. We have removed the website address "https://aqicn.org/map/chiang-mai/" from the abstract section as it is not appropriate to include URLs in this section. We have included the website address in the reference section instead. We apologize for any confusion this may have caused and appreciate your valuable feedback.

**Reviewer #3:** This is an important study that investigated the association between the air quality index outdoors and emergency visits for various health outcomes. These findings may contribute to policy change to improve outdoor air quality in Thailand in the future. The revision seemed to have accommodated comments from the reviewers. Additional comment is provided below.

The air quality data is collected outdoors while the location (indoor or outdoor) of the patient when the patient was transferred to ER is not clear. Most likely, the air indoors will not reflect the air outdoors due to building infiltration, filtration in HVAC systems, or the use of portable air cleaners. Can rerun your analysis by separating patients by their location when sent to ER? If this is not doable, can you explain why the air quality index outdoors can be used to hypothesize outdoor air pollution may be the cause of the ER visit?

**Answer:** Thank you for your additional comments and for bringing up this important point. We acknowledge that the location (indoor or outdoor) of the patient when they presented to the Emergency Department (ED) is not clear in our study. While it is true that air indoors may not reflect the air outdoors, we would like to clarify that all the air quality data used in our study were collected “outdoors”.

To address this concern, we have added the following statement to the methods section of the revised manuscript: "Daily outdoor AQI of PM2.5, PM10, ozone (O3), nitrogen dioxide (NO2), and sulfur dioxide (SO2) was collected from the Smoke Haze Integrated Research Unit (SHIRU) and the Air Pollution in Chiang Mai: Real-time Air Quality Index Visual Map website (https://aqicn.org/map/chiang-mai/). Notably, these pollution data were collected from the outdoor air quality.” This statement clarifies that our data was collected exclusively from outdoor sources.

While we were not able to separate patients by their locations when they presented to the ED, we hypothesize that the outdoor air quality index can be used as a proxy for outdoor air pollution. The relationship between outdoor air pollution and negative health outcomes has been well-established in several previous research.^1-7^ Our study adds to this body of research by investigating the association between the air quality index and specific health outcomes in the Chiang Mai region of Thailand.

We hope this explanation clarifies any concerns you may have had about the relationship between our air quality data and the health outcomes we investigated. We appreciate your feedback and valuable contributions to the peer review process.

Reference

1. Newby DE, Mannucci PM, Tell GS, Baccarelli AA, Brook RD, Donaldson K, et al. Expert position paper on air pollution and cardiovascular disease. European Heart Journal. 2015 Jan 7;36(2):83–93.

2. Rajagopalan S, Al-Kindi SG, Brook RD. Air Pollution and Cardiovascular Disease: JACC State-of-the-Art Review. Journal of the American College of Cardiology. 2018 Oct 23;72(17):2054–70.

3. Haikerwal A, Akram M, Del Monaco A, Smith K, Sim MR, Meyer M, et al. Impact of Fine Particulate Matter (PM _2.5_ ) Exposure During Wildfires on Cardiovascular Health Outcomes. Journal of the American Heart Association. 2015 Jul 17;4(7):1–10.

4. Shah AS, Langrish JP, Nair H, McAllister DA, Hunter AL, Donaldson K, et al. Global association of air pollution and heart failure: a systematic review and meta-analysis. The Lancet. 2013 Sep 21;382(9897):1039–48.

5. Xu Q, Li X, Wang S, Wang C, Huang F, Gao Q, et al. Fine Particulate Air Pollution and Hospital Emergency Room Visits for Respiratory Disease in Urban Areas in Beijing, China, in 2013. PLOS ONE. 2016;11(4):e0153099.

6. Jo YS, Lim MN, Han YJ, Kim WJ. Epidemiological study of PM_2.5_ and risk of COPD-related hospital visits in association with particle constituents in Chuncheon, Korea [Internet]. International Journal of Chronic Obstructive Pulmonary Disease. 2018 [cited 2019 Mar 31]. Available from: https://www.dovepress.com/epidemiological-study-of-pm25-and-risk-of-copd-related-hospital-visits-peer-reviewed-fulltext-article-COPD

7. Shah ASV, Lee KK, McAllister DA, Hunter A, Nair H, Whiteley W, et al. Short term exposure to air pollution and stroke: systematic review and meta-analysis. BMJ. 2015 Mar 24;350:h1295.
