# Supplementary material for: Association between air quality index and effects on emergency department visits for acute respiratory and cardiovascular diseases
Source: PLoS One. 2023 Nov 16;18(11):e0294107. doi: 10.1371/journal.pone.0294107 (PMC10653395; doi:10.1371/journal.pone.0294107)
Supplement: Attachment — Submitted filename: Response to reviewer PM2.5 T3.docx [file pone.0294107.s008.docx]

**Response to reviewer**

We would like to thank the editor and reviewers for their helpful suggestions. We have already revised the manuscript and hope that the revised manuscript has met the requirements for publication. Changes were track change in the revised manuscript.

Reviewer #1: Dear authors,

Thank you for your thoughtful revisions to the manuscript. Authors have revised the issues of concern that I outlined and have stated the study limitations that affect the interpretation of the results. I appreciate the effort the authors put into this work. The article is clearer, and this research is repeatable due to enough information you provide. I think your paper could be accepted for publication into PLOS ONE now.

**Answer: Thank you for your positive feedback and acknowledgement of the revisions made to the manuscript. We are glad that the article is now clearer and provides sufficient information for repeatability. We appreciate your consideration for accepting our paper for publication in PLOS ONE. We are grateful for the opportunity to contribute to the scientific community through this research, and we look forward to any further steps in the publication process.**

Reviewer #4: First of all, I would like to congratulate the authors for undertaking this research as it would add to the existing body of literature in the South and South-east Asian region on the topic of air quality and its impacts on health.

Major Comments:

• In general, the authors need to get the English corrected (sentence format) either by sending it to another colleague better in English or a native English language speaker. The authors can also use various freely available websites that can be used for English editing.

**Answer: Thank you for your suggestion. I have made the necessary revisions to improve the sentence structure and language with the assistance of a native English speaker.**

• There is a major confusion, as to what is the variable used by the authors for undertaking the study, PM2.5 concentration, AQI derived from PM2.5 or AQI derived from PM2.5/PM10 or AQI derived from a calculation using concentrations of PM2.5, PM10, ozone (O3), nitrogen dioxide (NO2), and sulfur dioxide (SO2).

**Answer: In our study, we exclusively utilized the AQI calculated from concentration.**

• Are there any adjustments done for any other physical / physiological aspects of the patients concerned for performing this study

**Answer: No adjustments were made for the physical or physiological aspects of the patients involved in this study.**

Minor Comments:

Abstract:

1. Pg 2: Result part of the abstract, “AQI of PM2.5 concentration was 89.0 ± 40.2”, this section is creating confusion if the value is a concentration or an index. Because concentration should be followed by a unit, but AQI could just be a value. The authors can mention in the beginning that AQI was derived from PM2.5 concentrations and then follow with only one terminology that is AQI. This would reduce a lot of the confusion.

**Answer: We apologize for the mistake. The sentence has been corrected as follows: “AQI of PM2.5 was 89.0 ± 40.2”**

2. In the abstract itself the authors can indicate if the association was positive or negative for the purpose of clarity.

**Answer: Thank you for your suggestion. We have revised the abstract to indicate the nature of the association for clarity.**

3. Could the mortality association be termed as pre-mature mortality. As this might not be the direct cause / only established cause.

**Answer: Thank you for your useful suggestion. We have revised the term "mortality with" to "unexpected deaths due to" to reflect that it may not be the sole established cause of mortality.**

Introduction

4. Pg 4: Please modify the following sentences “Air pollution is a major public health issue worldwide and represents the largest environmental problem, especially with harmful health effects.” As “Air pollution is a major public health concern worldwide and represents one of the largest environmental problems. 1”. Kindly check for these errors in the entire manuscript.

**Answer: We have revised the sentence as follows: "Air pollution is a major public health concern worldwide and represents one of the largest environmental problems." We will review the entire manuscript for similar errors.**

5. Pg 4: There are vague sentences that need significant modification i.e. “Thailand is a SEA country with exposure to high annual mean WHO Air Quality Guidelines (AQG).11,12”

**Answer: We have revised the sentence as per your suggestion. Here is the revised version: “Thailand experiences significant exposure to air pollution that exceeds the annual mean guidelines set by the WHO Air Quality Guidelines.”**

6. Kindly refrain from using such sentences “However, no previous study in SEA mentions the effects of PM2.5 on health impacts in ED visits”. There are quiet a few studies “IJERPH | Free Full-Text | The Effects of PM2.5 from Asian Dust Storms on Emergency Room Visits for Cardiovascular and Respiratory Diseases (mdpi.com) ; Short-term PM2.5 exposure and emergency hospital admissions for mental disease - ScienceDirect”

**Answer: Thank you for bringing this to our attention. We have revised the sentence as per your feedback. Here is the revised version: “However, few studies in SEA mentions the effects of PM2.5 on health impacts in ED visits”**

Methodology

7. Pg 5: The authors indicate “Daily outdoor AQI of PM2.5, PM10, ozone (O3), nitrogen dioxide (NO2), and sulfur dioxide (SO2)”. So how is the AQI calculated and what has been used. There is a strong confusion in terms of the AQI terminology used in Abstract and in the text.

**Answer: Thank you for your feedback regarding the calculation of the Air Quality Index (AQI) and the confusion surrounding its terminology in the abstract and text. We have edited the manuscript to provide clarification. Here is the revised version: “We examined the daily outdoor Air Quality Index (AQI) for pollutants including PM2.5, PM10, ozone (O3), nitrogen dioxide (NO2), and sulfur dioxide (SO2). Daily outdoor AQI of PM2.5, PM10, ozone (O3), nitrogen dioxide (NO2), and sulfur dioxide (SO2) The AQI serves as a standardized metric for assessing and communicating air pollution levels. It is calculated by converting the concentrations of these pollutants into sub-index values using established equations or algorithms. These sub-index values are then combined to derive the overall AQI value, providing an indication ofindicating the air quality level on a daily basisdaily. These were was collected from the Smoke Haze Integrated Research Unit (SHIRU) and the Air Pollution in Chiang Mai: Real-time Air Quality Index Visual Map website.”**

8. Pg 6: Again here, the authors indicate “The primary exposure variable was PM2.5”

**Answer: Thank you for your clarification. We have edited the sentence as follows: “The primary exposure variable was AQI of PM2.5”**

9. Pg 6: Another statement “Xt-q represented the focused AQI of air pollutants (PM2.5 or PM10) concentration”. That means were PM10 or PM2.5 concentrations used continuously for this study or randomly any particulate concentration was used.

**Answer: For this study, we specifically focused on the daily average Air Quality Index (AQI) of air pollutants. This indicates that AQI were used continuously throughout the study, rather than randomly selecting any particulate AQI. We have revised the sentence as follows: “Xt-q represented the focused daily average AQI of air pollutants (PM2.5 or PM10)”**

10. Following the four statements (three above and one in abstract), I am now in a confusion, what is the variable used by the authors for undertaking the study PM2.5 concentration, AQI derived from PM2.5 or AQI derived from PM2.5 or PM10 or AQI derived from a calculation using concentrations of PM2.5, PM10, ozone (O3), nitrogen dioxide (NO2), and sulfur dioxide (SO2).

**Answer: The variable used by the authors for undertaking the study is the PM2.5 AQI. The AQI derived in the study is based on a calculation that includes the concentrations of PM2.5, PM10, ozone (O3), nitrogen dioxide (NO2), and sulfur dioxide (SO2).**

Results and discussion

11. Pg7: 1st paragraph, the percentage of different cause specific respiratory diseases, is not coming up to 100%, kindly provide the breakup accordingly.
**Answer: Thank you for pointing out the issue. We have revised the sentence to provide a breakdown of the top three respiratory diseases leading to daily ED visits. Here is the revised version: “The top three respiratory diseases leading to daily ED visits were Upper Respiratory Tract Infections (28.5%), Pneumonia (27.3%), and Chronic Obstructive Pulmonary Disease (COPD) and Asthma (26%) respectively.”**

12. Confusing sentence “The AQI of all air pollutants was highest during April of both years (2018 and 2019), as shown in Supplementary Figure 2”. The current study period has only one April so why are the authors discussing about two Aprils.

**Answer: We apologize for the confusion caused by the sentence mentioning the AQI of all air pollutants being highest during April of both years (2018 and 2019). The current study period indeed covers only one April, and there was no intention to discuss two separate Aprils. The sentence will be revised to accurately reflect the findings of the study, specifying the highest AQI observed during the April of the study year. We have edit the sentence as follows: “The onset of the summer season heralded the culmination of peak levels in the AQI for all air pollutants. Specifically, this phenomenon was observed during the timeframe from April 2018 to March 2019, as demonstrated in Supplementary Figure 2.”**

Discussions

13. Please mention in complete sentences “This study found that AQI of PM2.5 was associated with the number of ED visits due”. What is the association?

**Answer: Thank you for your suggestion. We have revised the statement as follows: “This study found that the AQI of PM2.5 was associated with an increased number of ED visits due to ACS and** **pneumonia (lag days 1 and 2). Additionally, a significant association was observed between PM2.5 AQI and pneumonia on the current day and the following day after exposure.”**

Conclusions

14. Conclusion should be elaborated and given point wise for all the associations.

**Answer: We have carefully revised the manuscript to provide a more comprehensive and point-wise conclusion for all the associations observed in our study. Specifically, our revised conclusion now states: “In summary, our study provides robust evidence demonstrating the association between short-term PM2.5 pollution exposure and increased risks of ED visits for ACS and respiratory diseases, particularly pneumonia. These findings emphasize the need for effective measures to reduce PM2.5 pollution levels and protect vulnerable populations from the adverse health impacts of air pollution.”**
