# Supplementary material for: Association between air quality index and effects on emergency department visits for acute respiratory and cardiovascular diseases
Source: PLoS One. 2023 Nov 16;18(11):e0294107. doi: 10.1371/journal.pone.0294107 (PMC10653395; doi:10.1371/journal.pone.0294107)
Supplement: Attachment — Submitted filename: Response to reviewer 3 PLOS ver1.docx [file pone.0294107.s009.docx]

**Response to reviewer**

**We appreciate the editor's and the reviewers' insightful comments. The manuscript has already undergone revisions, and we are hopeful that the updated version satisfies the requirements for publication. Changes were tracks change in the revised manuscript.**

Reviewer #4: It is nice to see the revised version of the manuscript. While most of the comments are addressed well but one fundamental question needs more modification that the authors are not able to explain in a logical manner. Therefore, suggesting a major revision.

The response to the 3 different comments for the use of AQI are still not clear and creating confusion.
1. The terminology that should be used throughout the manuscript is only “AQI”

**Answer: Terminology: We apologize for any confusion caused by the inconsistency in AQI terminology used throughout the manuscript. We acknowledge the importance of using a standardized term consistently, and we will revise the manuscript to ensure that only "AQI" is used consistently in all sections.**

2. AQI is a calculation that uses values of all pollutant parameters to reach at one meaningful value. This is termed as AQI. Have the authors done it? Please provide the equation how AQI was calculated in this manuscript.

**Answer: AQI Calculation: We sincerely apologize for the oversight in not providing the equation for AQI calculation in the initial submission. In the revised version, we will include a detailed description of the AQI calculation method used in this study. The AQI was calculated based on the concentrations of PM2.5, PM10, ozone (O3), nitrogen dioxide (NO2), and sulfur dioxide (SO2). The precise formula used to calculate the AQI will be described in the supplementary section, which is displayed below this.**

**Daily air quality index calculation of each type of air pollution substance**

Calculated from the concentration of air pollution from the air quality measurement results with the level of air pollution concentration that is equivalent to the air quality index at various levels as follows (Table 1). Calculation of air quality index within the level is a straight line equation as follows.

I_p_ = [(I_HI_ - I_LO_)/(BP_HI_ - BP_LO_) * (C_p_ - BP_LO_)] + I_LO_

Where,

I_p_ = Index for pollutant p.

I_HI_ = AQI value corresponding to BP_HI._

I_LO_ =AQL value corresponding to BP_LO._

BP_HI_ = Breakpoint that is greater than or equal to C_p_.

BP_LO_ =Breakpoint that is less than or equal to C_p_.

C_p_ = Rounded concentration of pollutant p.

The highest index (highest value of I_p_) represents the AQI of the location.

**Table 1 Air pollution concentration equivalent to air quality index**

| **AQI Cluster** | **PM_2.5_ (µg./m^3^)** | **PM_10_ (µg./m^3^)** | **O_3_ (ppb)** | **CO (ppm)** | **NO_2_ (ppb)** | **SO_2_ (ppb)** |
| --- | --- | --- | --- | --- | --- | --- |
|  | **Average 24 hours continuously.** | | **Average 8 hours continuously.** | | **Average 1 hour** | |
| 0 - 25 | 0 - 25 | 0 - 50 | 0 - 35 | 0 - 4.4 | 0 - 60 | 0 - 100 |
| 26 - 50 | 26 - 37 | 51 - 80 | 36 - 50 | 4.5 - 6.4 | 61 - 106 | 101 - 200 |
| 51 - 100 | 38 - 50 | 81 - 120 | 51 - 70 | 6.5 - 9.0 | 107 - 170 | 201 - 300 |
| 101 - 200 | 51 - 90 | 121 - 180 | 71 - 120 | 9.1 - 30.0 | 171 - 340 | 301 - 400 |
| More than 200 | 91 and up | 181 and up | 121 and up | 30.1 and up | 341 and up | 401 and up |

µg./m3; Micrograms per cubic meter, ppb; parts per billion, ppm; parts per million

**Reference**

1. Air4Thai [Internet]. [cited 2023 Jul 22]. Available from: http://air4thai.pcd.go.th/webV2/aqi_info.php

2. Raj H, Vijaykumar S. A Study on Air Quality Index. ijcse [Internet]. 2019 [cited 2023 Jul 22];7:961–6. Available from: http://www.ijcseonline.org/full_paper_view.php?paper_id=3947

3. Refrain from mentioning “AQI of PM2.5 or AQI of PM10 because if that is the case (separately calculated) then why not directly use concentration

**Answer: We have verified that the source of Smoke Haze Integrated Research Unit (SHIRU) and the Air Pollution in Chiang Mai: Real-time Air Quality Index Visual Map website came form Thailand's air and noise pollution, the Pollution Control Department under the Ministry of Natural Resources and Environment at "aqmthai.com," provides only AQI values for each individual pollutant such as PM2.5, PM10, O3, NO2 and SO2. To eliminate any confusion, we will accurately state in the revised manuscript that our study used AQI values for PM2.5, PM10, O3, NO2, and SO2 separately.**

4. I am still confused, has the study used AQI of PM2.5 for further deep diving? Or it is AQI of PM10, AQI of NO2, AQI of O3 etc etc. separately or only AQI derived from calculation involving all pollutants together and not separately?

**Answer: In our study, we used the Air Quality Index (AQI) values derived from individual pollutant concentrations to assess their associations with health outcomes. Specifically, we obtained separate AQI values for PM2.5, PM10, O3, NO2 and SO2.**
