# Supplementary material for: Association between air quality index and effects on emergency department visits for acute respiratory and cardiovascular diseases
Source: PLoS One. 2023 Nov 16;18(11):e0294107. doi: 10.1371/journal.pone.0294107 (PMC10653395; doi:10.1371/journal.pone.0294107)
Supplement: Attachment — Submitted filename: Response to editor PM.docx [file pone.0294107.s010.docx]

Dear Editor,

I sincerely appreciate your consideration of my manuscript and the opportunity for revision.

Regarding your valuable feedback, I will make the following revisions:

1. Separate the Materials and Methods to "Data Collection" and "Data Analysis"

Answer: We separate the "Materials and Methods" section into two distinct sections, namely "Data Collection" and "Data Analysis”.

2. The DLNM equation is confusing. You have RH, temperature and AP, but the other variables were X_t. Why not write them to be consistent?

Answer: understand your concern about the inconsistency in variable notation within the DLNM equation. To address this, I will ensure that all variables are consistently represented as "X_t" throughout the equation, which will enhance the overall clarity and coherence of the mathematical formulation as revise as

‘$\log\left( E\left( Y_{t} \right) \right)=\alpha+ns\left( {RH}_{t},3 \right)+ns\left( Temperature,3 \right)+ns\left( {Focused\_AP}_{t},3 \right)+\sum_{i=0}^{q} \beta_{i}{(Focused\_AP)}_{t-i}+\varepsilon_{t}$

Where	$Focused\_AP\in\{AQI of PM_{2.5}, AQI of PM_{10}\}$ and

$Other\_AP\in\left\{ AQI of PM_{2.5}, AQI of PM_{10},AQI of O_{3},AQI of {NO}_{2} \right\}-\{Focused\_AP$}’

Thank you for your guidance and the opportunity to improve the manuscript. I will diligently work on these revisions and resubmit the manuscript accordingly.

Best regards,

Borwon Wittayachamnankul, MD, PhD

Department of Emergency Medicine, Faculty of Medicine, Chiang Mai University

Chiang Mai 50200, Thailand
